# Computational Modeling of a Low‐Cost Fluidic Oscillator for Use in an Educational Respiratory Simulator

**DOI:** 10.1002/anbr.202000112

**Published:** 2021-11-14

**Authors:** Tom Dillon, Caglar Ozturk, Keegan Mendez, Luca Rosalia, Samuel Dutra Gollob, Katharina Kempf, Ellen Tunney Roche

**Affiliations:** ^1^ Department of Mechanical Engineering Massachusetts Institute of Technology Cambridge MA 02139 USA; ^2^ Institute for Medical Engineering and Science Massachusetts Institute of Technology Cambridge MA 02139 USA; ^3^ Harvard‐MIT Program in Health Sciences and Technology Massachusetts Institute of Technology Cambridge MA 02139 USA; ^4^ Department of Mechanical Engineering Technical University of Munich Munich Germany

**Keywords:** computational fluid dynamics, education, fluidic oscillators, mechanical ventilation, respiratory

## Abstract

Herein, the computational modeling of a fluidic oscillator for use in an educational respiratory simulator apparatus is presented. The design provides realistic visualization and tuning of respiratory biomechanics using a part that is (i) inexpensive, (ii) easily manufactured without the need for specialized equipment, (iii) simple to assemble and maintain, (iv) does not require any electronics, and (v) has no moving components that could be prone to failure. A computational fluid dynamics (CFD) model is used to assess flow characteristics of the system, and a prototype is developed and tested with a commercial benchtop respiratory simulator. The simulations show clinically relevant periodic oscillation with outlet pressures in the range of 8–20 cmH_2_O and end‐user‐tunable frequencies in the range of 3–6 s (respiratory rate [RR] of 10–20 breaths per minute). The fluidic oscillator presented here functions at physiologically relevant pressures and frequencies, demonstrating potential as a low cost, hands‐on, and pedagogical tool. The model will serve as a realistic model for educating Science, Technology, Engineering, and Mathematics (STEM) students on the relationship between flow, pressure, compliance, and volume in respiratory biomechanics while simultaneously exposing them to basic manufacturing techniques.

## Graphical abstract

The computational modeling of a fluidic oscillator designed to convert continuous positive airway pressure (CPAP) machines into emergency mechanical ventilators is presented. The oscillator addresses potential ventilator shortages using a part that is inexpensive and easily manufactured. The fluid dynamics model is used to assess system performance, and a prototype is tested using a benchtop respiratory simulator.

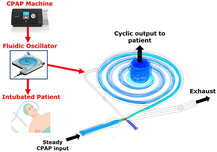

## 1 Introduction

Medical simulators have a wide array of applications, ranging from preclinical education to testbeds for medical devices and clinical training. For respiratory applications, one of the simplest and most common educational simulators is the bell jar model,^[^ ^1^, ^2^ ^]^ which consists of a pair of balloons (representing the lungs) enclosed in a bell jar (thoracic cavity) that is sealed at one end using a stretched piece of rubber (diaphragm). This model serves as a simple and cost‐effective educational tool to introduce preclinical students to the fundamentals of respiratory biomechanics. However, the model does not demonstrate the effect of dynamic physiological variables, such as respiratory rate (RR), peak inspiratory pressure (PIP), positive end expiratory pressure (PEEP), and lung health (which is coupled to lung compliance). More sophisticated respiratory simulators often used in clinical training (e.g., mannequin‐based simulators), allow tuning of dynamic respiratory variables, though these models are often too expensive for most preclinical classroom‐based settings (≈$150 000).^[^ ^3^, ^4^ ^]^ Moreover, clinical respiratory simulators often use a “black box” computational model to display the interactions between dynamic respiratory indicators, providing no physical visualization or intuition to the trainee as to the underlying biomechanics.^[^ ^3^, ^5^ ^]^

To improve upon existing physical respiratory demonstrative and training models whilst maintaining accessibility and cost, we propose the use of a fluidic oscillator to facilitate positive pressure mechanical ventilation of organic lungs and dynamic adjustment of respiratory variables. Distinct from our earlier work, where we actuated the diaphragm of a simulator with pneumatic artificial muscles, requiring a customized, and expensive electropneumatic control box,^[^ ^5^ ^]^ the oscillator fluidic controller presented herein is cheap, compact, and easily deployable in the educational environments, and only requires a simple, readily available air pump to provide continuous pressure. Fluidic oscillators are based on the bistable states of a jet of fluid inside a specifically designed flow chamber and can be harnessed to produce self‐excited oscillating fluid flow. In the case of our application, the fluidic oscillator converts a constant pressure air source at its inlet (provided by a standard pump or pressurized air supply) to an oscillatory output, simulating both inspiratory and expiratory flow.

The emergency ventilator, Automatic Respiration Management Exclusively for Emergencies (A.R.M.E.E), utilizes one such device, based on a fluidic oscillator developed by the US Army in 1965.^[^ ^6^ ^]^ Combined with a continuous positive airway pressure (CPAP) machine, fluidic oscillators can be used to create functional emergency use ventilators without the need for complex moving parts. However, the A.R.M.E.E. device is restricted to a maximum oscillation period of 1 s, which limits the minimum achievable RR, and is not physiologically realistic. In this article, we design, model, fabricate, and test an alternative fluidic oscillator design, capable of oscillating in physiological ranges of 3–6 s period (RR of 10–20 breaths per minute [bpm]) with output pressures in the range of 11–18 cmH_2_O, appropriate for recreation of realistic breathing biomechanics in organic swine lungs.

As an educational tool, our simulator provides a robust biomechanical mental model of breathing motion, illustrating the coupling between flow, pressure, volume, and compliance in a set of organic lungs. Moreover, a fluidic oscillator can also be designed to output a user‐tunable flow, providing students with an interactive experience and visual intuition of the effect of different breathing variables. Our design is easily implemented using simple fabrication techniques (e.g., 2D laser cutting), and requires no moving parts or electronics. We envision use of our design in school curricula to get children interested in STEM using commonly known materials and manufacturing methods. Crucially, we provide a much more accessible simulator alternative to the expensive mannequin‐based models, while maintaining a high‐fidelity simulation scenario. We envision that globally applicable teaching and visualization tools that appeal to students could revolutionize the K‐12 science classroom, inspiring the next generation of innovators in medicine and related fields.

## 2 Methods

**Figure 1:**
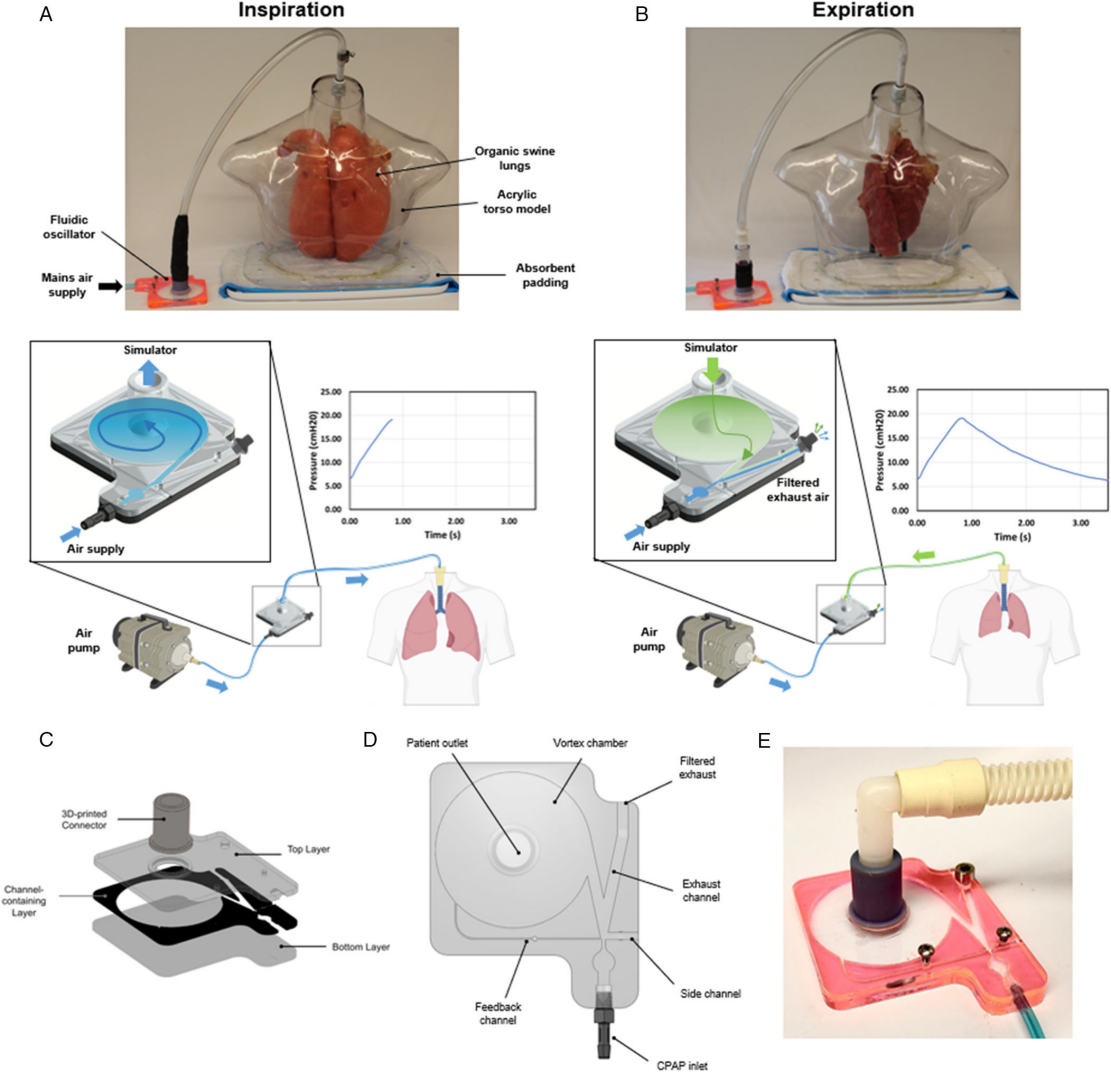
A low cost, rapidly deployable respiratory educational tool using a novel fluidic oscillator design. During inspiration, air flows from the high‐pressure air supply, through the oscillator, and into the organic swine lungs. A) Accumulation of air inside the lungs causes a corresponding increase in alveolar pressure. During expiration, flow reverses out of the lungs through the oscillator, exiting the system through a filtered exhaust. B) Deflation of the lungs results in a corresponding decrease in alveolar pressure. C) Oscillator assembly consisting of three layers of laser‐cut acrylic and a 3D‐printed connector. Plan view of the D) novel oscillator design, and E) final prototype including channel occlusion screws.

The overall concept for the educational simulator is shown in **Figure** **1**. For our design to be physiologically realistic, we targeted oscillation periods on the order of 3–6 s (RR 10–20 bpm) according to consensus guidelines for functional requirements for mechanical ventilators.^[^ ^7^ ^]^ The switching time for an oscillator is related to the characteristic time of the vortex chamber; that is, the time taken to fill the vortex chamber with fluid for a given mass flow rate

(1) $$\Delta t_{c}=\frac{\pi D^{2}h}{4v\dot{m}}$$

where $\Delta t_{c}$ is the characteristic time, *D* and *h* are the diameter and height of the vortex chamber, respectively, *v* is the specific volume of the fluid, and $\dot{m}$ is the mass flow rate.^[^ ^8^ ^]^ To improve upon the A.R.M.E.E. ventilator oscillation period of ≈1 s, we use a larger vortex chamber (88 vs 22 mm) and a central outlet to keep the vortex captive inside a chamber.^[^ ^6^ ^]^ The height of the vortex chamber is relatively small (2.4 mm), which serves to maintain adequate pressures at the patient outlet. An input flow rate of 30 L min^−1^ was selected based on standard ventilator specifications.^[^ ^7^ ^]^ Critical dimensions for the oscillator can be found in Figure S1, Supporting Information.

The nozzle‐diverter region exploits the Coanda effect (the tendency of a fluid to remain attached to walls), so that nozzle flow is not divided between the lungs and exhaust outlets throughout the respiratory cycle, but instead oscillates from one to the other.^[^ ^8^, ^9^ ^]^ The high‐velocity, low‐pressure air in the feedback channel (FC) pulls nozzle flow toward the vortex chamber during the inspiration (Figure 1A). This feedback loop serves the purpose of transporting a small portion of the outflow back to the nozzle‐diverting region to create the desired return flow behavior between the vortex chamber and exhaust channel (EC). At the end of inspiration, when the lungs are expanded, the pressure increase drives the flow switch at the nozzle‐diverter region from the vortex chamber (oscillator output) to the EC, initiating exhalation (Figure 1B). Air is pulled from the lungs, through the vortex chamber, and out through the EC, marking the exhalation phase of the respiratory cycle. The side channel (SC) pressure remains constant throughout the respiratory cycle, which provides more stable PIPs and PEEPs. The three set‐screws on the FC, SC, and EC are used to calibrate the oscillator (Figure 1C–E). We investigated key design parameters and used an iterative design process to optimize performance. This was carried out by adjusting parameters such as the vortex chamber height and diameter, and FC dimensions. We assessed the outcomes of these adjustments by both continuously monitoring key respiratory variables during simulation (PIP, PEEP, and RR), and qualitatively analyzing the corresponding 3D flow streamlines (e.g., switching behavior of the flow, vortex chamber turbulence, FC flow, etc.). A sample snapshot of these flow streamlines can be seen in Figure S2, Supporting Information.

ANSYS Fluent software was used to conduct a computational fluid dynamics (CFD) analysis of the oscillator. A *k*–*ε* turbulence model was selected, due to its capability of capturing adverse pressure gradients and turbulent dissipative effects.^[^ ^10^ ^]^ The mesh geometry contained more than 170 000 tetrahedral elements. A flow rate of 30 L min^−1^ was applied at the oscillator inlet, and the SC and EC outlets were set to atmospheric pressure. A no‐slip condition was defined at the oscillator walls. The convergence criterion for the residuals of mass, momentum, and energy equations was set to 10^−4^ for each simulation. The physical quantities such as pressure, flow rate, and velocity were also monitored for the convergence. An illustration of both the mesh and boundary conditions utilized can be found in Figure S3, Supporting Information.

A first‐order RC Windkessel model, which describes the lungs as a resistance element, *R*, connected in series with a capacitance element, *C*, was implemented to capture the variation in alveolar pressure over the respiratory cycle. The alveolar and pleural pressures, as well as the PEEP, are strongly coupled with the dynamics of the oscillator, as described in Equation (2):^[^ ^11^ ^]^

(2) $$P_{\text{alv}}(t)=\dot{Q}R+\frac{1}{C}\int_{0}^{\Delta t}Qdt+P_{\text{peep}}+P_{\text{pl}}$$

where *P* is pressure, *t* is time, *C* and *R* are lung compliance and resistance, respectively, and *Q* is flow, and the subscripts Alv, peep, and pl denote alveolar, PEEP, and pleural pressures, respectively (Figure S4, Supporting Information).

**Figure 2:**
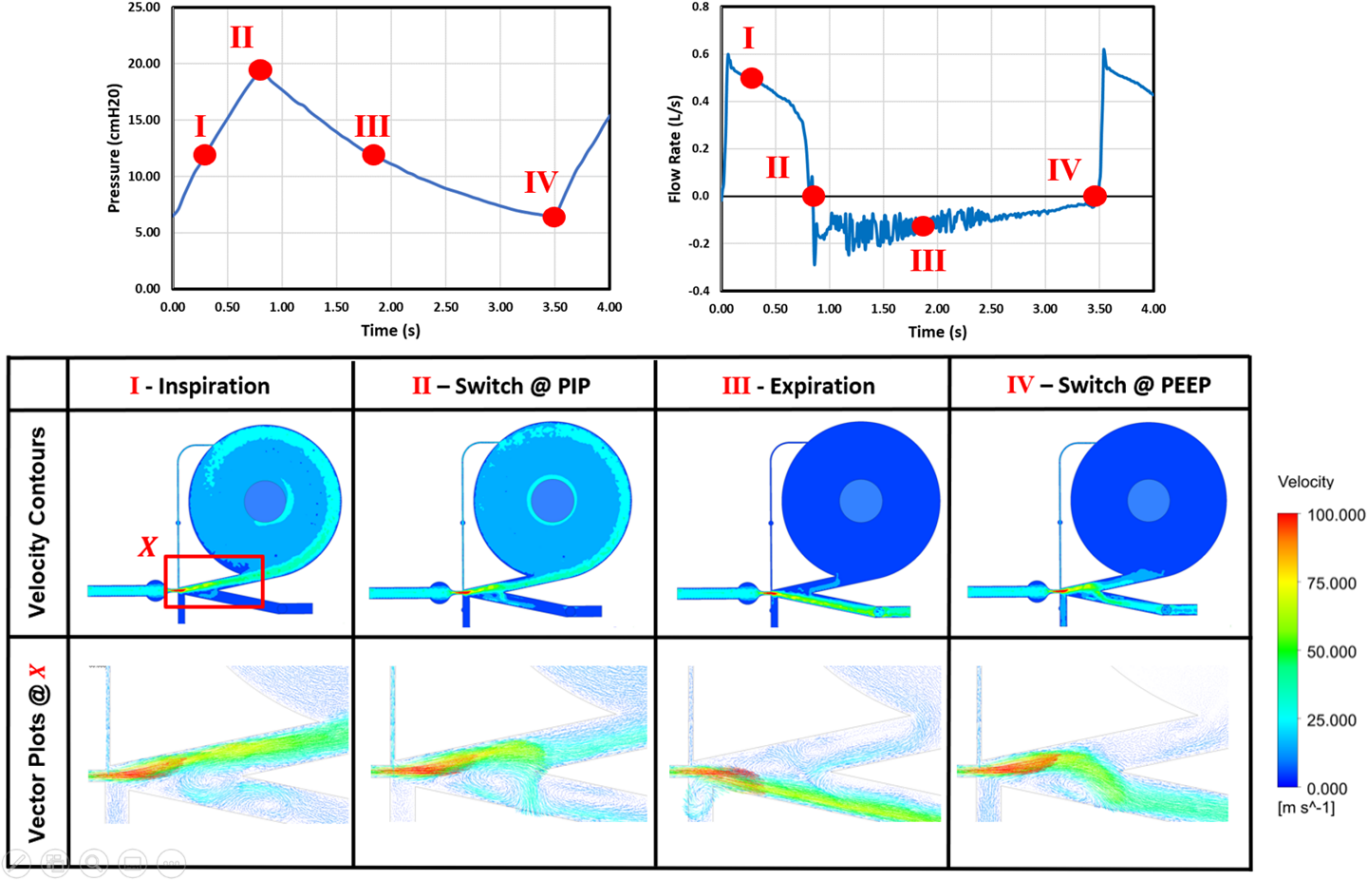
Velocity contours and vector plots (table), with associated pressure (graph, left) and flow rate (graph, right) profiles, taken at different stages throughout the respiratory cycle (I‐IV).

**Figure** **2** shows the CFD results at different stages throughout the respiratory cycle. At the beginning of inspiration, alveolar pressure is relatively low, and air circulates inside the vortex chamber before entering the lungs (Stage I). Eventually, high pressure stagnation inside the chamber causes nozzle flow to switch over to the exhaust side (Stage II). Following the switch, high pressure air is expelled from the lungs, returning along its inspiratory path through the oscillator and exits the system through the exhaust which a filter would be applied (Stage III). At the end of expiration, lung pressure is low, prompting flow to switch back to the patient inlet (Stage IV), beginning the respiratory cycle again.

**Figure 3:**
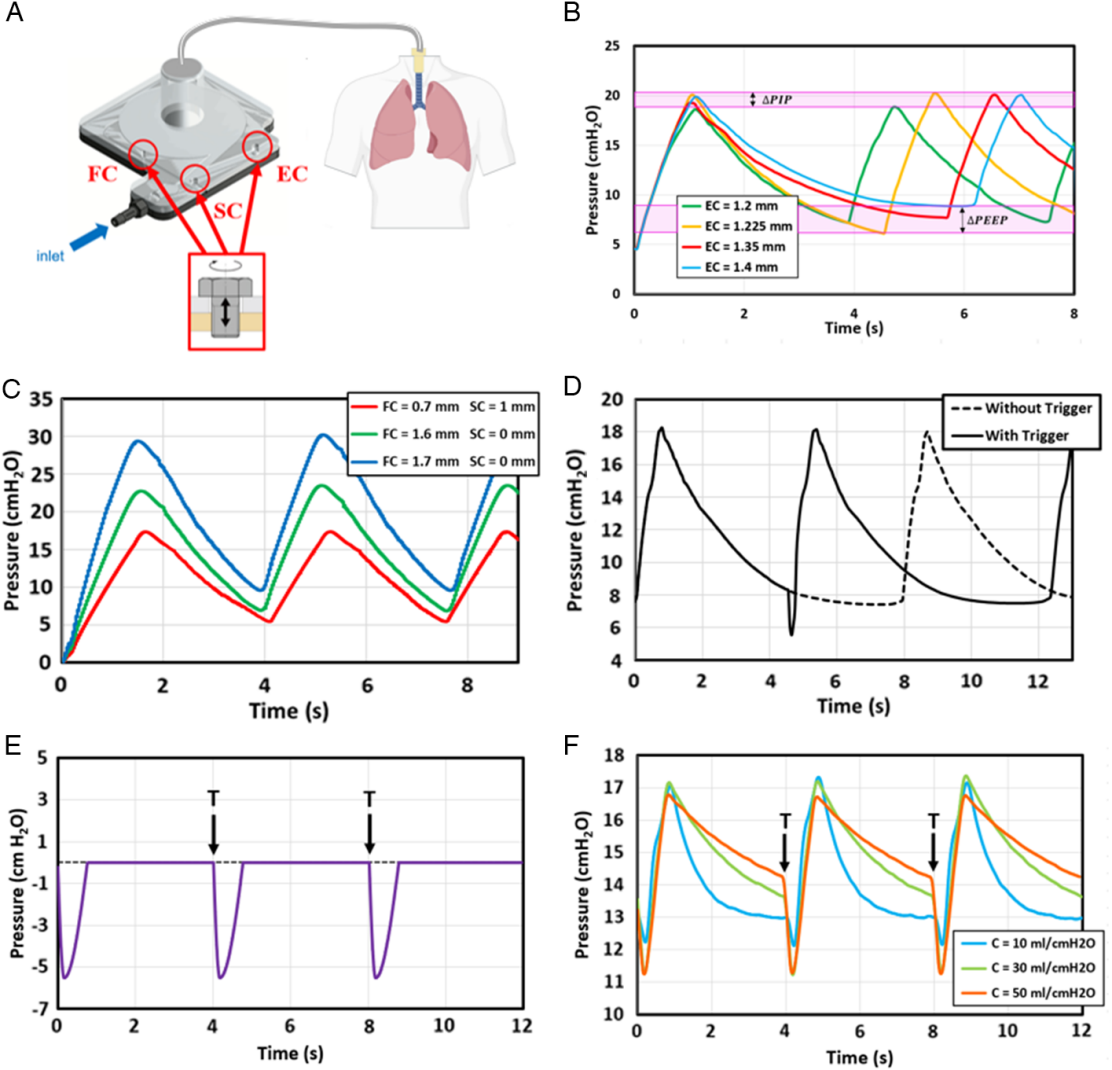
Functional parameter tuning A–D) in silico and E,F) in vitro. Illustration of the EC, FC, and SC screw locations referenced in (A). B) Variation in oscillation period for a selection of EC screw depths. C) △PEEP and △PEP ranges are indicated on the graph also. Tuning PIP and PEEP through simultaneous variation of FC and SC screw depth. D) Controlling the oscillation period in response to a patient trigger for mechanical ventilator simulation. Note the small decrease in pressure at ≈4.5 s representing a patient trigger. E) The muscle‐induced pressure profile applied by the Active Servo Lung (ASL) clinical lung simulator, simulating a patient trigger initiated at regular timepoints *T*. F) Corresponding experimentally measured pressure profiles for a range of lung conditions, with compliances ranging from 10 to 50 mL cmH_2_O^−1^.

An adjustable RR of 10–20 breaths per minute was identified as a key design criterion for the oscillator.^[^ ^7^ ^]^ A grub screw at the EC (EC, **Figure** **3**A) was implemented to allow tunability of the RR. The effect of EC screw depth on the oscillation period was studied in the CFD model. The other two set‐screws on the FC and SC were utilized to enable adjustments in PEEP and PIP. Visually, control of PIP varies the degree of inhalation the lungs experience (with higher values corresponding to larger tidal volumes or “deeper” breaths), whereas PEEP can vary the degree of exhalation (i.e., the amount of air in the lungs at the end of expiration). CFD analysis was conducted to determine the effects of the FC and SC depth on PIP (FC and SC, Figure 3A).

To demonstrate that our device is functional for a range of real‐world respiratory scenarios, we simulated an intubated set of lungs where positive pressure mechanical ventilation is provided by our oscillator prototype (in a similar fashion to the A.R.M.E.E. emergency ventilator setup).^[^ ^6^ ^]^ In this configuration, the diaphragm has a limited capacity to induce breathing effort, which could be caused by restrictive lung diseases that alter elastic compliance (e.g., pulmonary fibrosis, COVID‐19).^[^ ^12^, ^13^ ^]^ During mechanical ventilation, patient discomfort can arise when a delay exists between the desire to inhale, and the oscillator's switchover to inspiration.^[^ ^14^ ^]^ The patient's intention to inhale is detected in modern ventilators by a moderate decrease in lung pressure (i.e., a pressure trigger) induced by the diaphragmatic muscles.^[^ ^7^, ^15^, ^16^ ^]^ If the ventilator does not respond to this trigger, the patient will take a breath later than they are comfortable with. To represent the real‐world dynamics of pressure controlled mechanical ventilation using our educational simulator, we included the pressure drop induced by a standard patient trigger in our biomechanical model. The active pressure component contributed by the patient in this case is represented in Equation (2) by the pleural pressure, *P*_pl_. Here, the patient trigger is modeled as a forced step function of magnitude –3 cmH_2_O with first‐order dynamics and the time constant τ, defined as τ=*RC*.^[^ ^17^ ^]^ The effects of triggering on the oscillator's dynamics during the expiration phase were characterized, and the results are presented in the following sections.

## 3 Results and Discussion

Figure 3A–D shows the CFD results and functional parameter tuning in silico. The pressure profiles in Figure 3B–D show an inspiration‐to‐expiration ratio (*I*:*E*) that varies between ≈1:2 and ≈1:5, with clinically relevant PEEP and PIP values of 8 and 20 cmH_2_O, respectively.^[^ ^7^ ^]^ The demonstration of flow streamlines during the inspiration and expiration is shown in Figure S2, Supporting Information. Video S1, Supporting Information, shows the inspiration and expiration phases of the fluidic oscillator.

As shown in Figure 3B, increasing the EC screw depth reduces the rate of pressure decay between PIP and PEEP, facilitating an adjustable oscillation period of 3–5 s (RR 12–20 breaths per minute). The positive linear relationship was observed between exhaust screw depth, *d,* and oscillation period, *T* (Figure S5, Supporting Information). Variations in PIP and PEEP under different EC screw depths were found to be 1 and 3 cmH_2_O, respectively, which are small relative to the overall oscillation amplitude of 13 cmH_2_O. Hence, the EC screw allows for robust, predictable control of the oscillation period.

**Figure 4:**
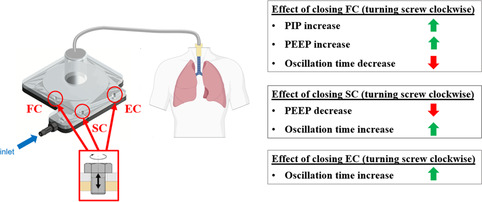
Summary of the key functions of the calibration screws. Arrows next to each bullet point illustrate positive (green arrow) or negative (red arrow) relationship between the number of clockwise screw rotations and the resulting effects.

Figure 3C shows that the simultaneous opening of the FC screw and closing of the SC screw increases PIP. The user can check the instructions and the information chart for details about calibration screws and incrementally adjust screw depths to achieve the desired PEEP and PIP outputs. As shown in **Figure** **4**, each calibration screw has a distinct impact on the respiratory characteristics. Both computational and experimental findings confirm these key functions of calibration screws during the respiratory cycle. Collectively, the EC, FC, and SC set‐screws allow the user to vary the degree of inhalation and exhalation of the lungs, as well as the frequency of respiratory motion, creating an interactive pedagogical experience.

Figure 3D shows that the oscillation period can vary dynamically from cycle to cycle based on a pressure trigger. Our data corroborate that a premature transition from the expiratory phase to the inspiratory phase is possible following a small decrease in alveolar pressure, representing a patient‐initiated breath when simulating mechanical ventilation. In the cycle immediately following the trigger, PIP, PEEP, and oscillation period all remain consistent.

Based on our computational results, a prototype was fabricated and tested using a clinical lung simulator (ASL 5000 Breathing Simulator) (Figure S6, Supporting Information). The use of a clinical lung simulator during the design process eliminated the need to procure organic lungs of varying elastic compliances, facilitating design verification for a range of lung pathologies. As shown in Figure 3E, the prototype is able to achieve stable, constant‐amplitude oscillations between (11.66 ± 0.37) cmH_2_O and (17.15 ± 0.33) cmH_2_O at the lung compliances tested (*C* = 10, 30, 50 mL cmH_2_O^−1^, representing pulmonary fibrosis, healthy lungs, and emphysema conditions, respectively).^[^ ^12^, ^13^ ^]^ with similar waveform shapes to those seen in silico, and showing minimal variation over a period of 60 s (*n* = 15 cycles for each waveform). Moreover, in our mechanical ventilation simulation, experimental results demonstrate that the prototype responds successfully to the patient trigger, as inspiration begins immediately following a patient‐initiated pressure drop by the lung simulator.

Figure 3E also shows that the prototype is functional for the range of lung compliances tested, while highlighting higher compliances produce a slower rate of pressure decay between PIP and PEEP. To counteract this effect, the user can simply vary the EC screw depth based on the instructions shown in Figure 4.

## 4 Conclusions

The main contributions of this work are as follows; (i) we present a low‐cost fluidic oscillator for use in an educational respiratory simulator (ii) CFD modeling demonstrated that our design is able to achieve physiologically relevant respiratory pressures and allows robust control of the RR, (iii) using a clinical simulator, we showed that a prototype of our design can successfully simulate mechanical ventilator over a wide range of lung compliances representing varying levels of lung pathology.

Given our aim was to design a cheap educational respiratory simulator, a key design criterion was simplicity. As such, we sought to avoid complex electromechanical systems that are expensive, potentially difficult to operate, and more prone to failure. Instead, we dedicated our efforts to understanding the oscillator's fluid dynamics using simulation and visualization in CFD. We provide an in‐depth explanation of the design's operation at each point throughout the respiratory cycle (see description of Figure 2). This knowledge in turn allowed us to better optimize the design, which is evident in the physiologically relevant RRs that are achievable (tunable oscillation period between 3 and 5 s).

To ensure our simulations most accurately depicted real‐world biomechanics of respiration, we coupled a first‐order Windkessel model of lung respiratory mechanics to our CFD model that can be adjusted for a range of COVID‐19 lung compliances. We provide further verification through implementation of a patient “trigger,” which is translated to an induced pressure drop in the Windkessel model. The effect of this trigger was also simulated experimentally, and similar results were obtained to those of the CFD. To our knowledge, these modeling approaches have not been applied to CFD simulation of fluidic oscillators to date.

However, there are some limitations associated with the current design. The slight discrepancy observed between the pressure oscillation obtained in silico (8–20 cmH_2_O) and in vitro (11–17 cmH_2_O) may be attributed to discretization errors associated with the meshing of the computational model. In addition, the idealized first‐order Windkessel model implemented in ANSYS may not represent complex second‐order resistive losses that would arise in a physical system. The ASL lung simulator utilizes a variable piston that moves under changes in the tidal volume, where both viscous and frictional losses could be present.

While this study was focused on the educational aspects, future work could also be extended to clinical training, providing a realistic testbed for practicing thoracic interventions. Complex interventional strategies, such as bronchoscopies and intubation, can be difficult for students to comprehend initially. Simulating the mechanics of the respiratory system in a low‐cost, tunable model could provide a hands‐on learning tool to aid the development of a visual mental model of specific interventional strategies. Clinicians in the medical field may also prefer to use our design for interventional simulations over more sophisticated alternatives, given the apparatus does not require knowledge of complex control systems and is easy to setup.

In summary, this work introduces a low cost, rapidly deployable respiratory educational tool using a novel fluidic oscillator design. The design facilitates precise, independent control of of PIP, PEEP, and oscillation period using simple dials, allowing students in the K‐12 classroom and beyond to experiment with respiratory variables and visualize results in real time. Finally, the operational concept of the oscillator was validated by the prototype testing, and PEEP and PIP values between 11 and 17 cmH_2_O were achieved. A summary of the primary simulation and experimental results is shown in Table S1 (Supporting Information). Further tuning of the prototype may be necessary to extend this range to the 8–23 cmH_2_O as seen in the simulation. Further research may also explore the full range of design parameters (e.g., FC, SC, and EC screw depth) that determine the oscillator's functional performance.

Overall, we believe our model will enhance understanding and intuition of respiratory physiology and pathology in a visual and interactive manner, presenting a hands‐on learning opportunity to STEM classrooms globally. Further, we envision that compact, low cost, tunable fluidic oscillators, designed based on CFD simulations will have utility for actuation of other types of anatomic simulators (e.g., cardiac), dynamic assist devices for dynamic organs, and more broadly to other types of soft robots that require oscillating pressure waveforms for their actuation.

## 5 Experimental Section

5.1

5.1.1

##### Prototype Fabrication

A prototype of the oscillator was developed as consisting of three layers of laser‐cut scratch‐ and UV‐resistant cast acrylic sandwiched together. The central layer (2.4 mm thickness) contained the main oscillator geometry, and the top and bottom layers (3.2 mm thickness) were sealed using clear fast cure epoxy (J‐B Weld). A 3D‐printed connector was attached to an opening in the top layer above the vortex chamber, which was in turn connected to the ASL 5000 Breathing Simulator. An additional three openings were cut above the FC, SC, and EC channels, and screws for variable occlusion were placed into each (Figure 3E). These screws were used to alter flow resistance as predicted in silico. 3D computer aided design files of oscillator design are openly available in Supporting Information to allow for easy replication of our fabrication process in the classroom.

##### Prototype Testing Setup

Tests on the respiratory simulator—ASL 5000 Breathing Simulator (IngMar Medical)—were conducted with a constant input flow rate of ≈30 L min^−1^ measured using a digital mass flow meter (SFM300, Sensirion). Pressures and volumes were recorded directly by the ASL simulator. To simulate mechanical ventilation, A muscle (diaphragmatic) pressure profile simulating a patient‐initiated breath every 4 s was applied in the ASL settings. To simulate the respiratory mechanics of emphysema, pulmonary fibrosis, and healthy lungs, simulator compliance was varied between 1 and 50 mL cmH_2_O^−1^.^[^ ^12^, ^13^, ^18^ ^]^ The airway resistance and trigger amplitude were chosen to align with those used in the in silico model (*R* = 3 cmH_2_O/L/s and 5.5 cmH_2_O, respectively).^[^ ^12^, ^13^, ^19^ ^]^

##### Statistical Analysis

The results were reported as mean values ±SD in the main text and Table S2, Supporting Information. Cycles number (*n*) was 15 for each waveform.^[20]^ Data processing was carried out using Matlab software (Mathworks Inc., MA, USA).

## Conflict of Interest

The authors declare no conflict of interest.

## Data Availability Statement

The data that support the findings of this study are available from the corresponding author upon reasonable request.

## Supporting information

Supplementary Material

Supplementary Material
