# Supplementary material for: Computational Modeling of a Low‐Cost Fluidic Oscillator for Use in an Educational Respiratory Simulator
Source: Adv Nanobiomed Res. 2021 Nov 14;1(12):2000112. doi: 10.1002/anbr.202000112 (PMC7995041; doi:10.1002/anbr.202000112)
Supplement: Supplementary file 1 — Supplementary Material [file ANBR-1-0-s002.pdf]

Supporting Information

Title

Computational fluidic modeling of a low-cost fluidic oscillator for conversion of a CPAP machine into an emergency use mechanical ventilator

*Author(s), and Corresponding Author(s)**

*Tom Dillon†, Dr. Caglar Ozturk†, Keegan Mendez, Luca Rosalia, Samuel Dutra Gollob, Katharina Kempf, Prof. Ellen T Roche**

†These authors contributed equally to this work and are co-first authors

*Prof. Ellen T Roche
MIT E25-344, 77 Massachusetts Avenue, Cambridge, MA 02139
E-mail: etr@mit.edu

Keywords: Computational Fluid Dynamics, COVID-19, Fluidic Oscillator, Mechanical Ventilation

Computational Fluid Dynamics *(CFD) Simulation Method*

ANSYS Fluent 17.0 software was used to model the flow field inside the oscillator. A time-dependent transient solver was used to perform unsteady flow simulation. The air was modeled as an incompressible fluid with a density of 1.225 kg/m^3 and a dynamic viscosity of 1.7894×10^{-5} kg/m-s. During the grid generation process, adequate orthogonal quality, aspect ratio, and the skewness were targeted as per guidelines offered in the ANSYS Fluent User's Guide.[9] Turbulence was simulated with the k-ε model, and a mean $\gamma+ < 3$ value with appropriately sized cells near domain walls. The total cell count was approximately 250,000 cells. The first-order discretization method was used for the pressure and first-order upwind discretization method

used for momentum calculations. The inlet boundary condition was set to 30 L/min flow rate, the exhaust outlet pressure was set to 0 Pa, and the patient outlet pressure was coupled with the 2-element Windkessel model. Lung compliance and resistance were modeled as C = 30 ml/cmH_2O and R = 3 cmH_2O/L/s respectively.

Oscillator Parameters

The oscillator comprises three adjustable screws located at side channel (SC), feedback channel (FC), and exhaust channel (EC). Before beginning ventilation, the three calibration screws should be adjusted to set PIP, PEEP, and I:E ratio. Pressure-controlled ventilation can be initiated after this calibration, and should be tuned for both patient breath-triggered ventilation and time-triggered ventilation PIP and PEEP can also be adjusted based on CPAP flow rate. Flow rates from 30-50 L/min allow for ranges of PEEP and PIP between 8-15 cmH_2O and 15-30 cmH_2O respectively.

Oscillator Calibration Methodology

1. Adjust calibration screws (FC, SC and EC) to the fully open position (by turning counter-clockwise).
2. Connect the output of the oscillator to the patient ventilation tubing.
3. Attach and turn on CPAP. Set inflow to 30 L/min.
4. Slowly close the FC (clockwise) until a steady cycle with a PIP of approximately 15-20 cmH_2O is achieved.
5. Slowly close the SC (clockwise) to adjust the PEEP to approximately 10 cmH_2O.
6. Slowly close the EC (clockwise) to adjust the I:E ratio.

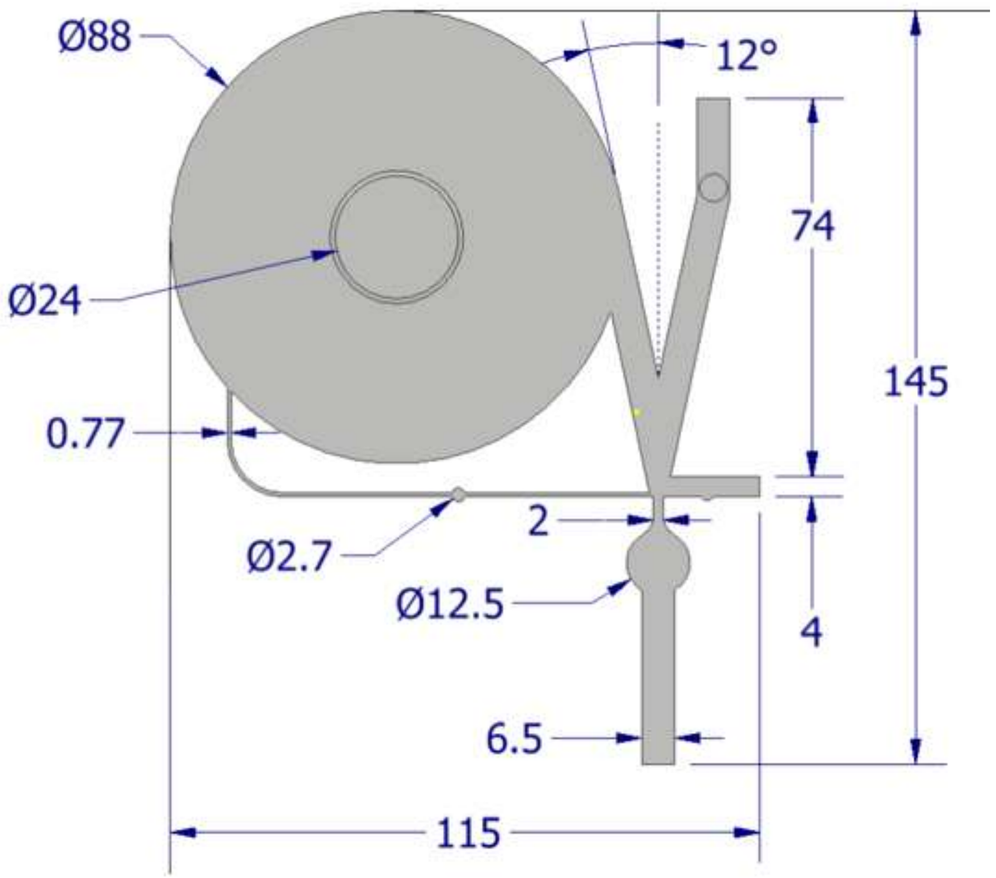

Figure S1. Critical dimensions of the main oscillator geometry (all scalar measurements in mm).

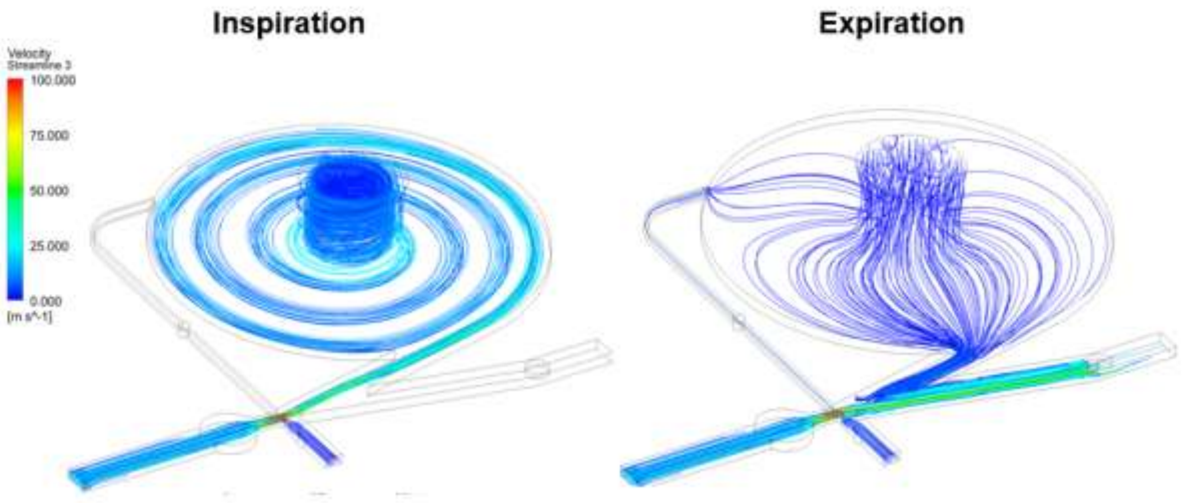

Figure S2. Streamline velocity results for the oscillator during inspiration and expiration, marked respectively as I and III in Figure 2.

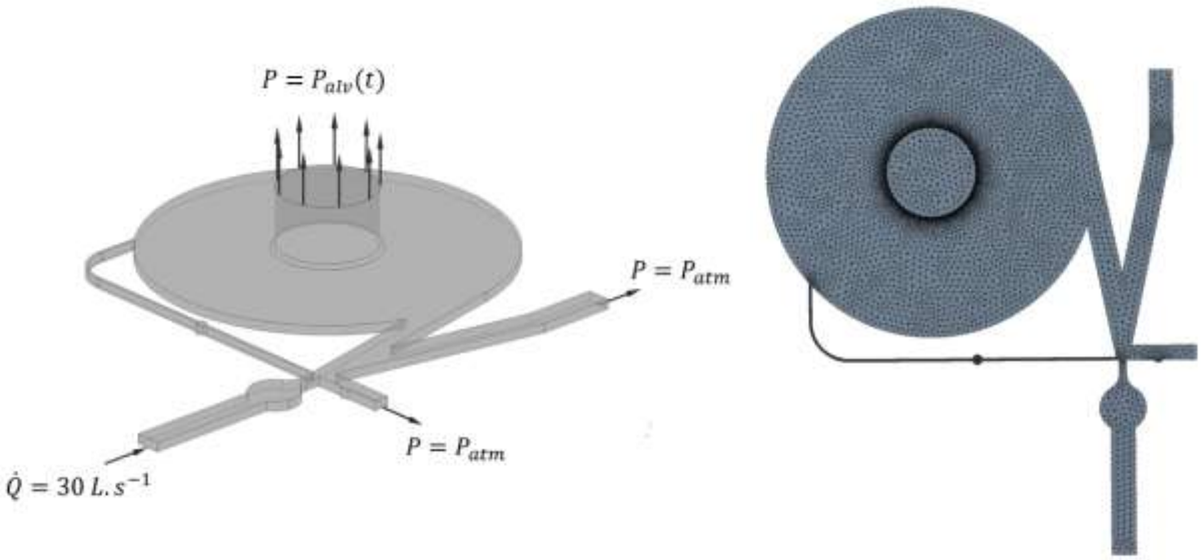

Figure S3. Boundary conditions (left) and mesh (right) applied to the fluidic domain in the CFD model.

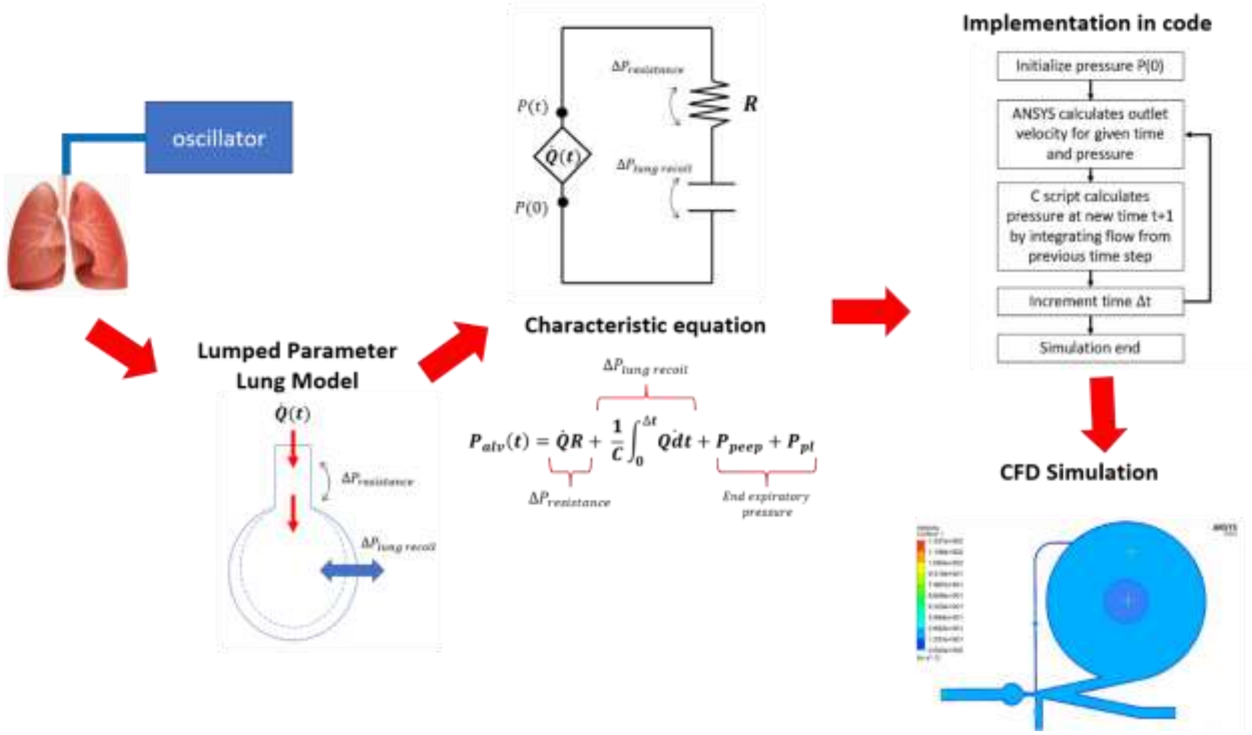

Figure S4. Simulation workflow using the 2-element Windkessel model.

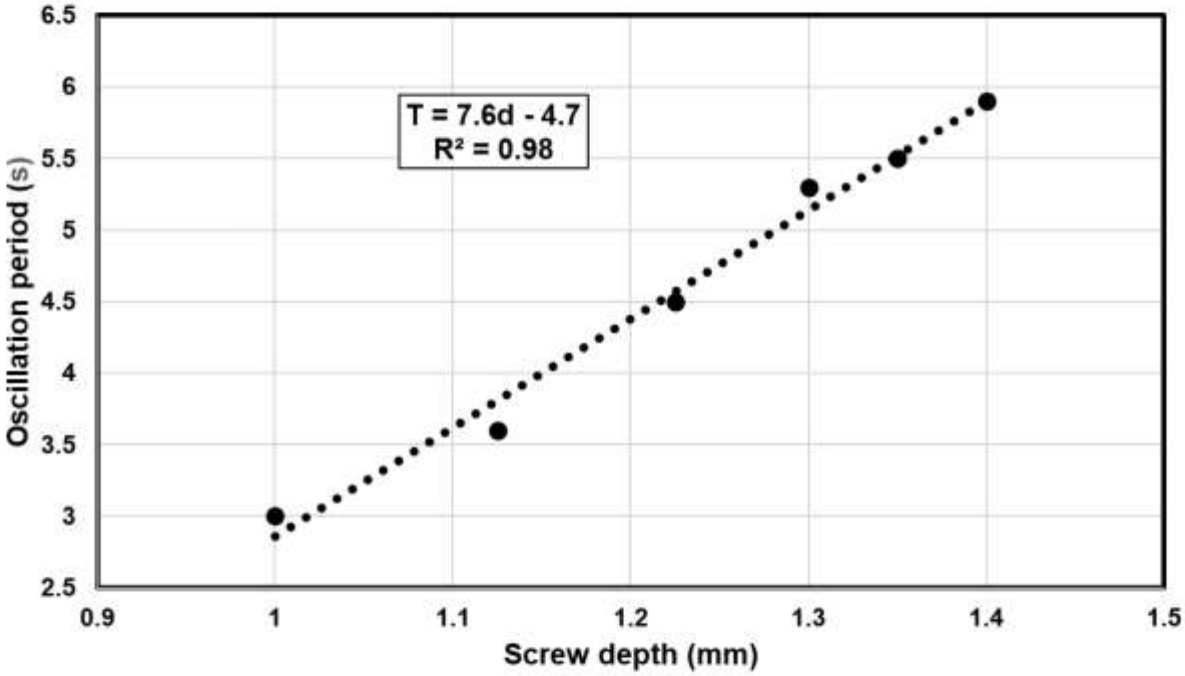

Figure S5. Variation in the oscillation period based on the Exhaust Channel (EC) screw depth.

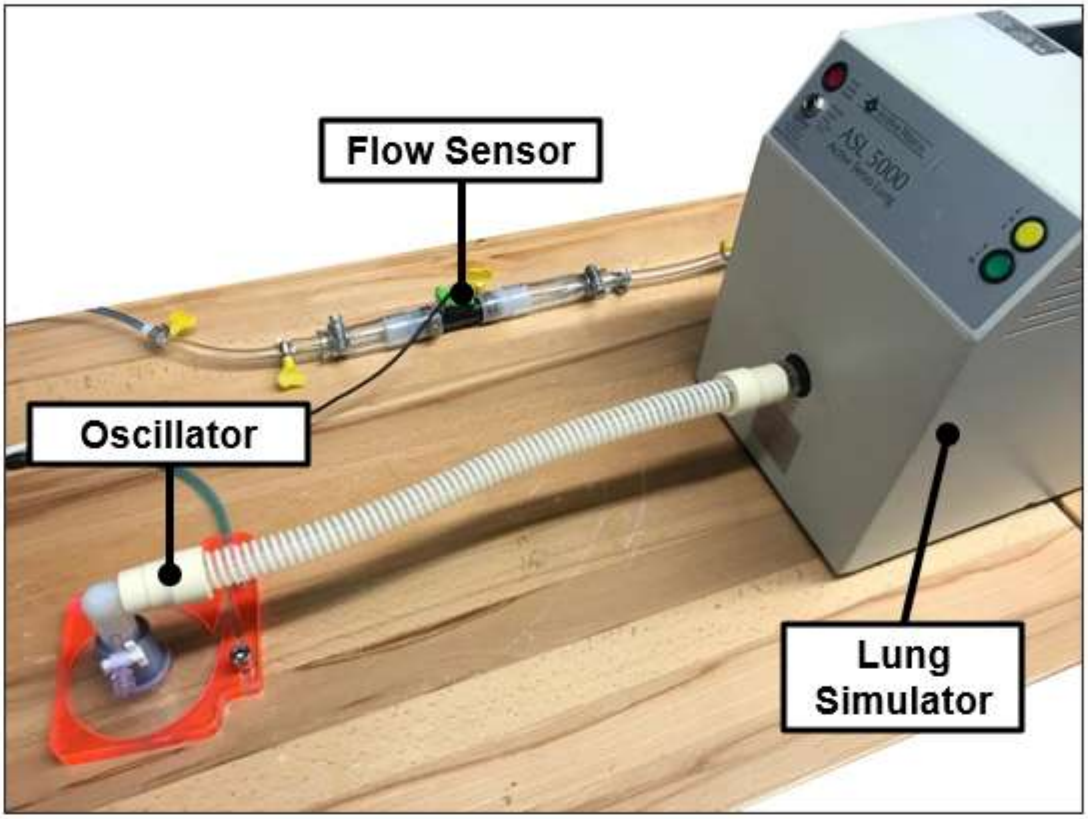

Figure S6. Simulator testing setup

Table S1. Summary of targeted values, simulation results and experimental results.

	**Target**	**Simulation**	**Experimental**
PEEP [cmH_2O]	6-10	6-10	11-13
PIP [cmH_2O]	20-23	18-28	16-18
RR [bpm]	10-20	8-20	15-20
I:E	1:1-1:3	1:2-1:5	1:2.5-1:5

Table S2. Summary of peak inspiratory pressure (PIP), positive end-expiratory pressure (PEEP), and respiratory rate (RR) at different lung compliances.

Compliance (ml/cmH_2O)	PIP (cmH_2O)	PEEP (cmH_2O)	RR	n
10	17.12 ± 0.18	12.13 ± 0.10	14.98 ± 0.02	15
30	17.55 ± 0.07	11.58 ± 0.05	14.99 ± 0.02	15
50	16.79 ± 0.06	11.26 ± 0.05	14.99 ± 0.02	15
